# Detoxification of Ochratoxin a by *Weizmannia coagulans* CGMCC 9951: Characterization, Mechanism, and Application in *Cornus officinalis* Pulp

**DOI:** 10.3390/toxins18050194

**Published:** 2026-04-22

**Authors:** Cuiping Shao, Yalin Li, Ying Wu, Lina Zhao, Pingping Tian, Shaobin Gu

**Affiliations:** 1 College of Food and Bioengineering, Henan University of Science and Technology, Luoyang 471023, China; 2 Henan Engineering Research Center of Food Microbiology, Luoyang 471023, China; 3 National Demonstration Center for Experimental Food Processing and Safety Education, Luoyang 471023, China; 4 Henan Provincial Key University—Enterprise Joint R&D Center of Probiotics Scientific Evidence-Based Research and Industrial Application, Luoyang 471023, China

**Keywords:** ochratoxin A, *Weizmannia coagulans* CGMCC 9951, biological degradation, enzymatic mechanism, *Cornus officinalis* pulp

## Abstract

This study investigates the degradation characteristics, pathways, and mechanisms of ochratoxin A (OTA) by *Weizmannia coagulans* CGMCC 9951 (*W. coagulans* CGMCC 9951), as well as its detoxification effect on *Cornus officinalis* pulp through fermentation. The strain efficiently degraded 300 ng/mL of OTA within 72 h (98% degradation) under optimal conditions of 37 °C, pH 5.0, and 180 rpm. Active degradation substances were primarily localized in the cell-free supernatant (CF). The degradation activity was significantly inhibited by heat treatment, proteinase K, EDTA, Cu^2+^, and organic reagents, suggesting an enzymatic mechanism. UHPLC-MS and MS/MS analysis indicated that OTA appears to be degraded to a product consistent with ochratoxin α (OTα). Based on homology to known OTA-degrading carboxypeptidases, the gene encoding WGU28473.1 was selected, expressed in *E. coli*, and confirmed to possess OTA-degrading activity. Molecular docking suggested potential interactions between the enzyme and OTA. Under optimal conditions, co-fermentation with *Cornus officinalis* pulp contaminated with 300 ng/mL OTA for 96 h resulted in a 74% degradation of OTA. The fermentation process increased the pulp’s sugar content and ABTS^+^ free radical scavenging capacity, reduced acidity, and improved the safety of the pulp. These findings demonstrate that *W. coagulans* CGMCC 9951 efficiently degrades OTA and improves pulp quality, highlighting its potential as a starter culture for detoxifying OTA-contaminated food.

## 1. Introduction

Ochratoxin A (OTA), produced by certain species of *Aspergillus* and *Penicillium* [1], is a toxic isocoumarin metabolite with harmful effects like nephrotoxicity, hepatotoxicity [2], immunotoxicity [3], teratogenicity, carcinogenicity, mutagenicity, and genotoxicity [4,5]. Its stable, heat-resistant nature makes it challenging to eliminate, posing severe health risks even at low concentrations [6]. Notably, the Joint FAO/WHO Expert Committee on Food Additives has established a provisional tolerable weekly intake (PTWI) of 100 ng/kg body weight, corresponding to approximately 14 ng/kg body weight per day, highlighting the high sensitivity of renal endpoints to OTA exposure [7]. In addition, regulatory limits have been established in different regions; for example, the European Commission has set maximum OTA levels at 3 μg/kg for cereals and 5 μg/kg for roasted coffee. The International Agency for Research on Cancer classifies OTA as a Group 2B carcinogen [8], prompting increased research into controlling its contamination [9]. Although OTA contamination is mainly associated with cereals, coffee, and dried fruits, increasing evidence indicates that medicinal herbs are also susceptible during cultivation, processing, and storage [10,11]. Fruit-derived medicinal materials are particularly vulnerable due to their nutrient-rich composition and susceptibility to fungal colonization [12,13]. *Cornus officinalis*, a fruit-derived medicinal material recently approved as a food–medicine dual-use substance, is increasingly used in functional foods [14]. Therefore, investigating OTA degradation in *Cornus officinalis* pulp is of practical relevance for ensuring the safety of such products. Current methods for removing or degrading OTA include physical (adsorption), chemical (oxidation), and microbiological (biodegradation) approaches [15]. Physical adsorption using materials such as bentonite and chitosan can bind OTA but can also lead to nutrient loss and saturation [16,17]. Oxidation processes, including photocatalysis and ozone treatment [18], effectively degrade OTA but may lead to nutrient loss and pollution [19]. Biodegradation, utilizing microbes or enzymes, is increasingly favored for its cost-effectiveness and minimal environmental impact [20] converting OTA into non-toxic substances without harming nutrients. Moreover, its compatibility with food processing systems significantly enhances its practical application in industrial settings.

Many bacterial and fungal strains have been documented to degrade OTA, including *Lysobacter* sp. CW239 [21], *Brevundimonas naejangsanensis* ML17 [22], *Stenotrophomonas acidaminiphila* CW117 [23], *Cytobacillus oceanisediminis* CO29 [18], *Brevibacillus* sp. ALJ02 [24], *Aspergillus niger* [25], and *Pleurotus ostreatus* [16]. The OTA degradation mechanisms involved amide bond hydrolysis, dechlorination, and lactone ring opening, collectively resulting in the formation of various dihydroisocoumarin derivatives [26]. Among these, the hydrolysis of amide bonds, which yielded the non-toxic compound OTα, was considered the most effective detoxification pathway [27].

In addition to these traditional microorganisms, probiotics in OTA degradation has also gained widespread attention. Some researchers have found that certain *lactic acid bacteria* can not only convert malic acid to lactic acid through fermentation, but also reduce OTA levels. The degradation product was confirmed as OTα via LC–MS/MS [28]. *Short-branching Bacillus*, isolated from cheese, is a typical smear microorganism in dairy production. This bacterium not only enhances the aroma of dairy products but also transforms OTA in dairy into the non-toxic OTα and L-phenylalanine [29]. Other *Brevibacterium* species, such as *B. antiquum*, *B. auranticum*, *B. casei*, *B. iodinum*, and *B. linens*, which can be safely applied in fermented foods, have shown OTA-degrading potential [30].Additionally, a probiotic (YL-1) isolated from soil, which has high OTA elimination efficiency, produces the non-toxic OTα as the degradation product [31]. Therefore, probiotics show great potential for both research and practical applications.

*W. coagulans* CGMCC 9951 possesses an excellent safety profile and demonstrates robust environmental adaptability, as well as the inherent metabolic capacity to degrade ochratoxin A (OTA). These attributes render it a highly promising candidate for addressing mycotoxin-related food safety challenges. This study investigated the OTA-degrading characteristics and mechanisms of *W. coagulans* CGMCC 9951. To assess its practical application potential, the strain was applied to a complex plant matrix, *Cornus officinalis* pulp, which serves as a model substrate that more accurately reflects the conditions found in real herbal and food products. The results aimed to provide viable microbial resources and technical support for the biological detoxification of OTA in food systems.

## 2. Results and Discussion

### 2.1. Growth Curve and OTA Degradation Kinetics of W. coagulans CGMCC 9951

**Figure 1:**
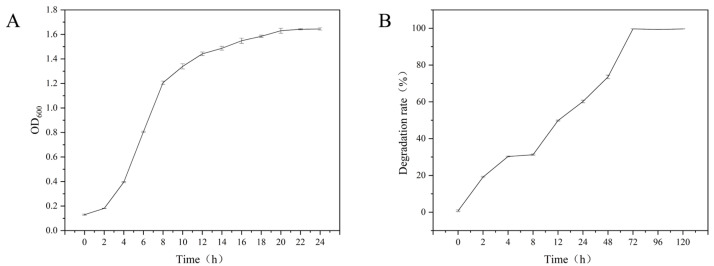
Growth and Degradation characteristics of OTA by strain *W. coagulans* CGMCC 9951. (**A**) Growth curve of *W. coagulans* CGMCC 9951; (**B**) Time course of OTA Degradation by *W. coagulans* CGMCC 9951 (*p* < 0.05).

The growth curve and OTA degradation dynamics of *W. coagulans* CGMCC 9951 are shown in Figure 1. The strain has a short lag phase (0 h–2 h), an exponential phase (2 h–12 h), and enters a stationary phase after 12 h (Figure 1A). During the early growth stage, the OTA degradation rate was limited. A degradation rate of approximately 30% was observed at 4 h, which may be partly attributed to rapid adsorption or binding of OTA to the cell surface [32]. Meanwhile, OTA may also act as an inducer [20], stimulating the expression of OTA-degrading enzymes in *W. coagulans* CGMCC 9951. After 72 h (Figure 1B), OTA degradation reaches 98%, indicating substantial OTA-degrading capacity. A five-day growth assay showed that when the bacterial density stabilized (Supplementary Figure S1), the OTA degradation rate remained high without significant change, demonstrating that OTA degradation by this strain is a stable and essentially irreversible process.

### 2.2. Effect of Fermentation Conditions on the OTA Degradation Activity of W. coagulans CGMCC 9951

**Figure 2:**
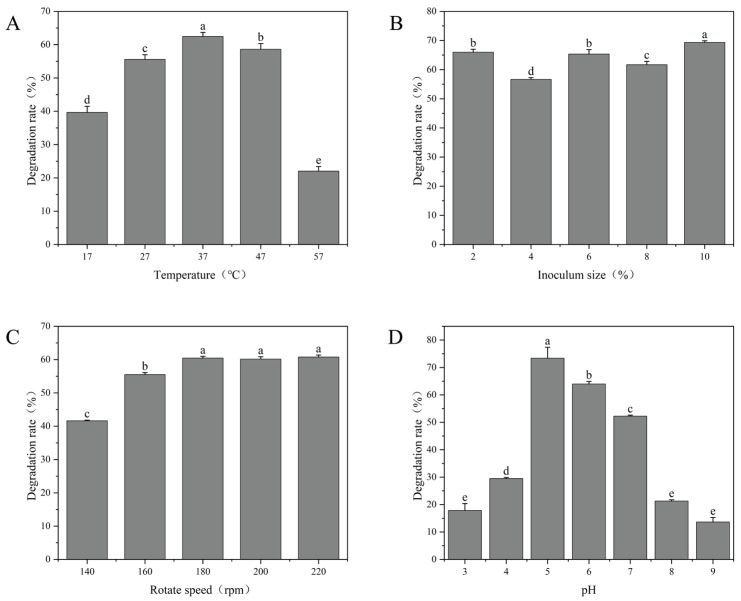
Degradation rates of *W. coagulans* CGMCC 9951 under different fermentation conditions. (**A**) Temperature; (**B**) Inoculum size; (**C**) Rotate speed; (**D**) pH. Different lowercase letters indicate significant differences among groups (*p* < 0.05).

The effects of temperature, inoculum size, agitation speed, and initial pH on OTA degradation by *W. coagulans* CGMCC 9951 were evaluated. As shown in Figure 2A, OTA was degraded most effectively at 37 °C with degradation rates above 60%. When the temperature was raised to 57 °C, the rate sharply reduced to 22%. This trend is consistent with previous studies on OTA degradation by *Saccharomyces cerevisiae* [33], indicating that temperature plays a significant role in microbial OTA degradation.

The inoculum size had a certain effect on the OTA degradation rate (Figure 2B), but the degradation rate at 2% inoculum was already at a relatively high level, possibly because this inoculum size provided an optimal initial cell density for the strain. At the end of fermentation, the final microbial populations in the 2%, 4%, 6%, 8%, and 10% inoculation groups were approximately 1.21 × 10^9^ CFU/mL, 1.3 × 10^9^ CFU/mL, 1.56 × 10^9^ CFU/mL, 1.5 × 10^9^ CFU/mL and 1.9 × 10^9^ CFU/mL, respectively. Rotation speed exerted a more pronounced influence. Increasing the speed from 140 rpm to 180 rpm raised the degradation rate from 42% to 61% (Figure 2C). This improvement is likely attributable to higher dissolved oxygen levels or greater bacterial contact with OTA. Overall, the strain demonstrated effective OTA degradation over a broad range of rotation speeds (180 rpm–220 rpm).

The initial pH was also a critical factor in OTA degradation. As shown in Figure 2D, the OTA degradation rate increased sharply from pH 4.0 to 5.0 and maintained above 70% at pH 5.0 but decreased dramatically at more acidic or alkaline pH values. This aligns with the findings of Cho et al., who reported that *Aspergillus tubingensis* also exhibited peak degradation activity at pH 5.0 [34]. Uninoculated controls at each pH confirmed that OTA was chemically stable under the experimental conditions (37 °C, 24 h), with OTA recovery of 95.4 ± 1.4% across all tested pH values (Supplementary Figure S2), indicating that the observed degradation was attributable exclusively to microbial activity.

### 2.3. Effect of OTA Initial Concentration on the Growth and Degradation Rate of W. coagulans CGMCC 9951

**Figure 3:**
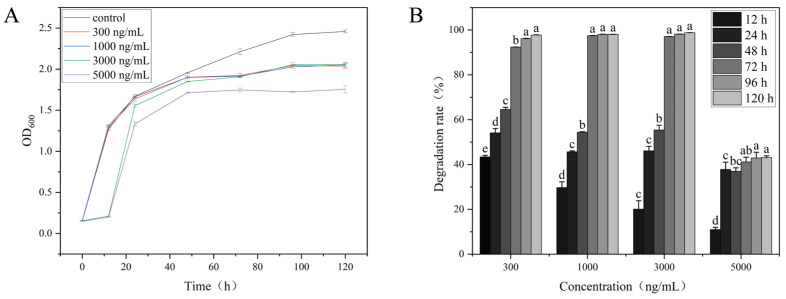
Effect of initial OTA concentration on growth and degradation rate of *W. coagulans* CGMCC 9951. (**A**) Growth curve; (**B**) Degradation rate. Different lowercase letters indicate significant differences among groups (*p* < 0.05).

As shown in Figure 3, OTA significantly inhibits the growth rate and degradation capacity of *W. coagulans* CGMCC 9951, with the effects being both time and dose dependent. In the control group, The OD_600_ increased gradually over time and eventually plateaued, indicating normal bacterial growth. During the initial stages of incubation (12 h and 24 h), OD_600_ values of the low-concentration treatment groups were similar to those of the control group. However, as the incubation progressed, both the *W. coagulans* CGMCC 9951 of growth and degradation rates in the high-concentration (5000 ng/mL) OTA group decreased significantly, likely due to the toxic effects of OTA, which disrupt normal metabolic processes [35]. The low-concentration OTA group displayed milder effects, though its growth remained lower than that of the control group. A similar trend was reported in *Acinetobacter pittii* AP19, where OTA inhibited growth despite efficient degradation, indicating a metabolic trade-off between detoxification and biomass accumulation [20].

### 2.4. Analysis of Active Fraction in W. coagulans CGMCC 9951 Degrading OTA

**Figure 4:**
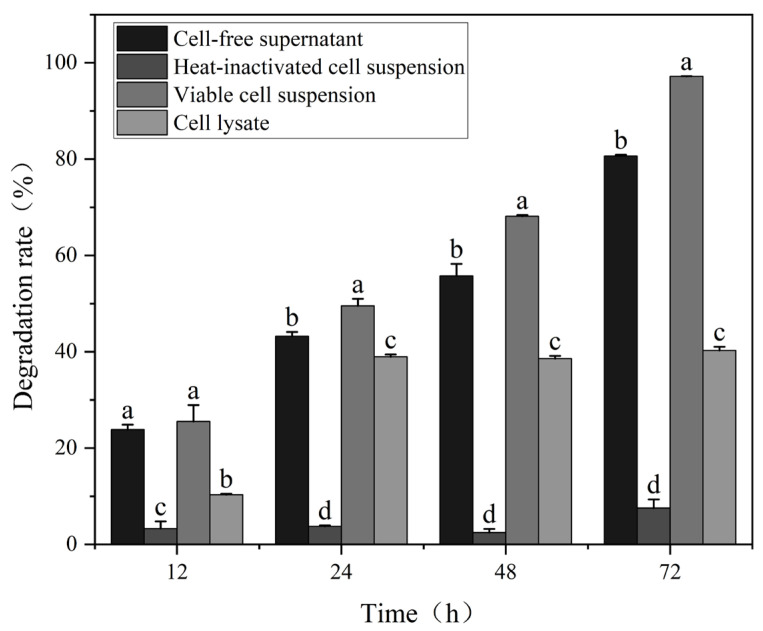
Degradation rates of various components by *W. coagulans* CGMCC 9951. Different lowercase letters indicate significant differences among groups (*p* < 0.05).

Generally, biological detoxification mechanisms involve both cellular adsorption and degradation processes [36]. As shown in Figure 4, the CF, VC, HI and CL exhibited large differences in their degradation rates, notably, CF and VC showed a significant increase over reaction time. The VC degradation rate reached 97% after 72 h, The high OTA degradation activity of the VC in PBS buffer is likely attributable to the pre-existing degrading enzymes accumulated in and on the cells during prior cultivation, as enzymatic catalysis does not require active cell growth or new enzyme synthesis. This is consistent with previous findings in *Acinetobacter pittii* AP19, where the viable cell suspension completely removed OTA within 6 h despite being resuspended in buffer without growth components [20]. By contrast, the HI was only 7%.

The removal ratio of the CF was lower than that of VC, but extending the reaction time to 72 h, the degradation rate of the CF reached 81%. These results showed that the CF can effectively remove the OTA after 72 h of reaction, indicating that the OTA-degrading ability of *W. coagulans* CGMCC 9951 was mainly attributed to biodegradation rather than cell adsorption.

The CL showed limited OTA degradation, consistent with reports that bacterial OTA-degrading enzymes are mainly extracellular or periplasmic [37]. Intracellular activity is inherently low, as seen in *Acinetobacter* sp. neg1 (~33% degradation by cell lysate) [38]. Ultrasonic disruption may further reduce activity due to heat and radical-induced protein denaturation. As is well known, the degradation of OTA is facilitated by the combined action of multiple enzymes [39]. There may be multiple active degradation substances present in *W. coagulans* CGMCC 9951.

### 2.5. Stability Study of CF from W. coagulans CGMCC 9951

**Figure 5:**
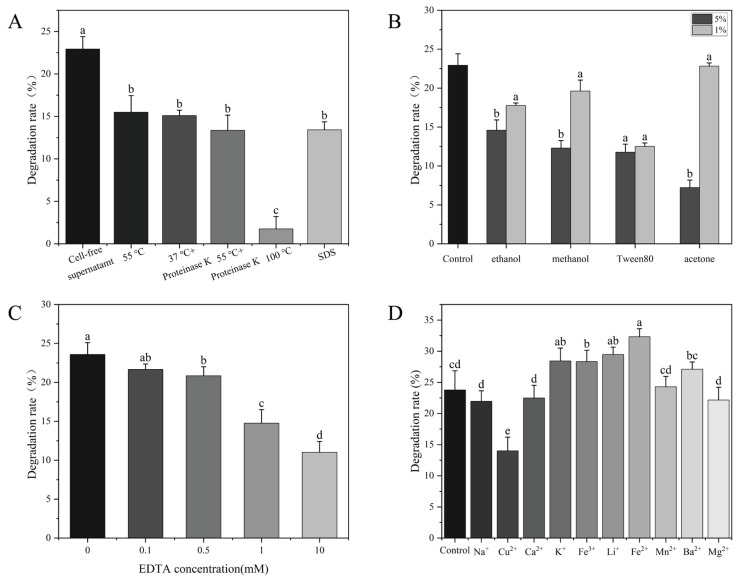
Stability of *W. coagulans* CGMCC 9951 fermentation supernatant. (**A**) Heat, proteinase K, and SDS; (**B**) Organic solvents; (**C**) EDTA; (**D**) Metal ions. Different lowercase letters indicate significant differences among groups (*p* < 0.05).

As shown in Figure 5A, boiling the CF for 30 min nearly completed destroyed the OTA degradation activity. In contrast, incubation of the CF at 55 °C for 1 h resulted in a 34% loss of degradation activity. In addition, OTA degradation was reduced 43% by treatment of the CF with 1 mg/mL proteinase K at its optimal temperature of 55 °C for 1 h. Notably, proteinase K could not degrade OTA [20]. Treatment with 50 mg/mL SDS for 1 h also reduced the ability of CF to degrade OTA. These results strongly indicated that the factor responsible for OTA degradation is an enzyme. Organic solvents also inhibited OTA degradation in a dose-dependent manner [18]. Among the organic solvents tested, acetone (5%) had the strongest inhibitory effect on the OTA degradation activity of CF (Figure 5B).

As shown in Figure 5C, treatment with 0.1 mM, 0.5 mM, and 1 mM EDTA reduced the OTA degradation rate from 24% to 20%. Notably, OTA degradation activity decreased significantly when EDTA concentration was increased to 10 mM, indicating that metal ions play a crucial role in the strain’s OTA degradation [40]. Therefore, we studied the effects of different metal ions on OTA degradation. As shown in Figure 5D, K^+^, Li^+^, and Fe^2+^ significantly improved the OTA-degrading activity, Mg^2+^ significantly decreased the degradation of OTA [41]. These results indicated that the OTA degradation is mediated by an enzyme, likely a metalloenzyme.

### 2.6. Identification of OTA Degradation Products

**Figure 6:**
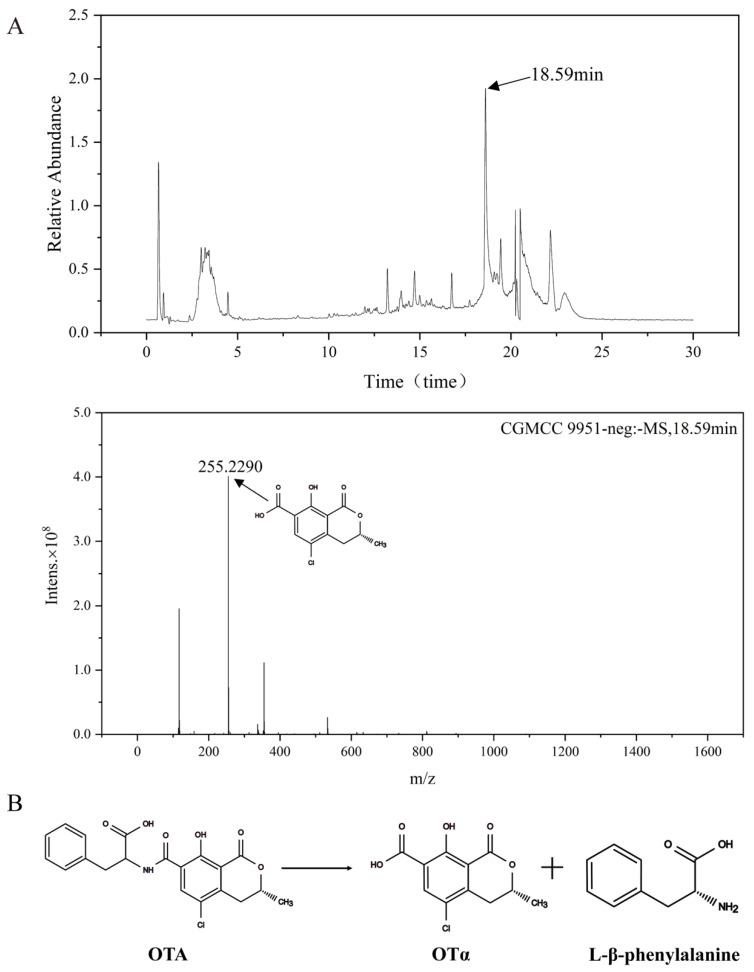
Peak plots of OTA degradation products detected by UHPLC-MS. (**A**) Total ion chromatogram and the first-order mass spectrum of the degradation product. The peak at 18.59 min corresponding to the degradation product; (**B**) The OTA degradation pathway.

The degradation product resulting from the *W. coagulans* CGMCC 9951 acting on OTA was analyzed via UHPLC-MS. Based on mass spectrometry analysis, the product was inferred to be OTα [42]. As shown in Figure 6, the peak observed at a retention time of 18.59 min exhibited the same mass transition characteristics as OTα, with a molecular ion [M−H]^−^ at *m*/*z* 255.2290. To further support the structural assignment, MS/MS analysis of the precursor ion was performed (Supplementary Figure S3). A characteristic fragment at *m*/*z* 211.0045 ([M−H−CO_2_]^−^, 44 Da) was observed [43]. The 255 → 211 transition and the neutral loss of CO_2_ are consistent with the fragmentation behavior of OTα [44]. This suggests that amide bond hydrolysis may be involved in the degradation process. Although L-β-phenylalanine is the expected co-product of amide bond hydrolysis alongside OTα, it was not detected in the present study. This is consistent with previous reports in which phenylalanine was also absent from the degradation medium, as the released amino acid is likely rapidly assimilated or further catabolized by the bacterial cells [38]. Indeed, phenylalanine is a common carbon and nitrogen source for bacteria and is efficiently metabolized via the phenylacetate pathway [45]. Given that multiple enzymes may synergistically contribute to OTA degradation in this strain, further studies are needed to elucidate the exact enzymatic mechanism. Notably, OTα, the primary degradation product of OTA, has been shown to be non-toxic in previous studies [15].

### 2.7. Hydrolysis of OTA by Recombinant WGU28473.1

**Figure 7:**
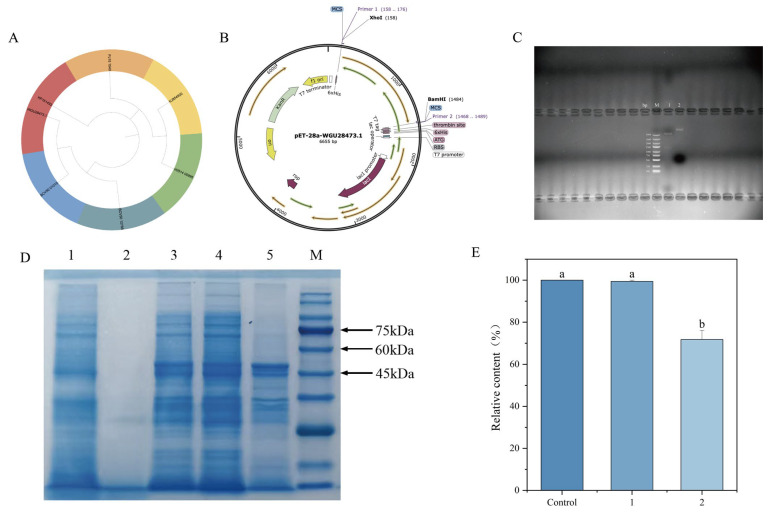
OTA hydrolysis by recombinant WGU28473.1 (**A**) Adjacency phylogenetic tree based on the amino acid sequences of carboxypeptidase (WGU28473.1) and other OTA hydrolases; (**B**) Recombinant plasmid map; (**C**) pET-28a-WGU28473.1 digestion by Xho I and BamH I (M, DNA marker; lane 1, digested pET-28a-WGU28473.1; lane 2 circular pET-28a-WGU28473.1; bp, base pairs) (**D**) SDS-PAGE of the recombinant WGU28473.1: Lane M, molecular weight marker; Lane 1, cell lysate of *E. coli* BL21 (DE3)-pET-WGU28473.1 without IPTG; Lane 2, culture supernatant of *E. coli* BL21 (DE3)-pET-WGU28473.1 induced with IPTG (after cell removal by centrifugation); Lane 3, cell lysate of *E. coli* BL21 (DE3)-pET-WGU28473.1 induced with IPTG; Lane 4, supernatant of *E. coli* BL21 (DE3)-pET-WGU28473.1 induced with IPTG (after centrifugation); Lane 5, precipitation of *E. coli* BL21 (DE3)-pET-WGU28473.1 induced with IPTG (after centrifugation). Color arrows indicate the positions of protein bands corresponding to molecular weights of 75 kDa, 60 kDa, and 45 kDa; (**E**) Degradation rate of OTA and crude enzyme: 1, crude enzyme of *E. coli* BL21 (DE3)-pET-WGU28473.1 without IPTG; 2, crude enzyme of *E. coli* BL21 (DE3)-pET-WGU28473.1 with IPTG. Different lowercase letters indicate significant differences among groups (*p* < 0.05).

Several microbial carboxypeptidases have been identified as enzymes involved in OTA biodegradation [20]. These metallopeptidases are known to hydrolyze the amide bond of OTA, converting it into the non-toxic compound OTα, and are characteristically heat-sensitive and susceptible to inhibition by proteinase K [46]. Given this, we surveyed the coding sequence (CDS) of *W. coagulans* CGMCC 9951 for homologous carboxypeptidases. A D-ala-D-ala carboxypeptidase, encoded by MKO99_00365 (WGU28473.1), was identified as a promising candidate. As shown in Figure 7A, WGU28473.1 falls into the same phylogenetic clade as KP161493 from *Bacillus amyloliquefaciens* [47], a carboxypeptidase previously reported to degrade OTA. Sequence alignment revealed that WGU28473.1 exhibits 91% coverage and 59% amino acid identity with KP161493. This structural similarity suggests a possible functional correlation, warranting further experimental validation. To investigate whether WGU28473.1 possesses OTA-degrading activity, the corresponding gene was ligated into the pET-28a (+) vector and expressed in *E. coli* BL21 (DE3) (Figure 7B). The double digestion (Xho I and BamH I) verified that recombinant plasmid pET-28a-WGU28473.1 was successfully constructed (Figure 7C). As shown in Figure 7D, the induced protein was detected in the soluble supernatant, and the molecular weight of the enzyme was approximately 50 kDa, consistent with the theoretical value. When the induced crude enzyme was incubated with 300 ng/mL OTA, significant degradation activity was observed, whereas no change in OTA content was detected with the non-induced control (Figure 7E). After incubation of the purified protein (0.3 mg/mL) with 300 ng/mL OTA for 1 h, the OTA content decreased to 46%. The results of enzyme purification and the degradation assay are provided in the Supplementary Materials (Supplementary Figures S4 and S5).

### 2.8. Binding Model of OTA and WGU28473.1

**Figure 8:**
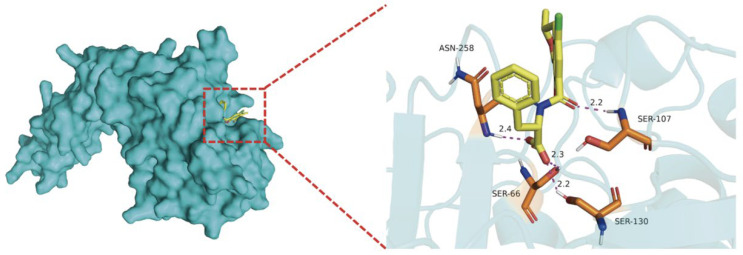
The 3D binding model of WGU28473.1 with OTA. In the **left** panel, blue represents the protein receptor, and yellow represents the OTA ligand; In the **right** panel, the red dashed lines represent hydrogen bonds.

To investigate the interaction between WGU28473.1 and OTA, molecular docking was performed using AutoDock Vina (Version 1.2.6). Both the WGU28473.1 and OTA structures were converted into PDBQT format using AutoDock Tools (Version 1.5.6). As shown in Figure 8, the binding free energy was −8.4 kcal/mol, indicating strong binding between WGU28473.1 and OTA. In addition, multiple hydrogen bonds were formed between OTA and protein residues (ASN 258, SER 66, SER 130, and SER 107), which may contribute to stabilizing the WGU28473.1-OTA complex. These results suggest that WGU28473.1 can bind OTA with high affinity, supporting its potential involvement in OTA degradation.

### 2.9. Exploration of W. coagulans CGMCC 9951 Application in Cornus officinalis Pulp

**Figure 9:**
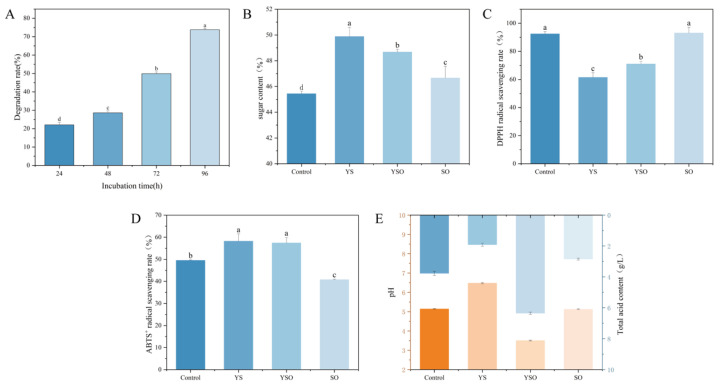
Fermentation performance of *W. coagulans* CGMCC 9951 in *Cornus officinalis* pulp. (**A**) OTA degradation rate in fermented *Cornus officinalis* pulp by *W. coagulans* CGMCC 9951; (**B**) Polysaccharide content; (**C**) DPPH radical scavenging rate; (**D**) ABTS^+^ radical scavenging rate; (**E**) pH and total acid content. Different lowercase letters indicate significant differences among groups (*p* < 0.05).

As shown in Figure 9A, after 96 h of fermentation of *Cornus officinalis* pulp with *W. coagulans* CGMCC 9951, the OTA content in the pulp decreased by 74%. This result suggested that *W. coagulans* CGMCC 9951 demonstrates good adaptability within the pulp of *Cornus officinalis*. Following probiotic fermentation (YS group), the pulp’s sugar content increased (Figure 9B) [48]. This increase may be attributed to the hydrolysis of structural polysaccharides in the protoplasts of *Cornus officinalis* by hydrolytic enzymes, such as cellulase and pectinase, secreted by *W. coagulans* CGMCC 9951 during fermentation. Importantly, the presence of OTA did not significantly affect the protoplasts’ sugar content.

Antioxidant capacity plays a key role in maintaining metabolic balance within organisms [49]. As shown in Figure 9C,D, the DPPH radical scavenging activity decreased, while the ABTS^+^ radical scavenging activity increased by 8% after fermentation of the pulp. This enhancement may result from the fermentation process by *W. coagulans* CGMCC 9951, which increased the free polyphenol content in the pulp, thereby improving its antioxidant activity [24].

After fermentation with *W. coagulans* CGMCC 9951, the pH of the *Cornus officinalis* pulp increased, while total acidity decreased from 3.77 to 1.92 (Figure 9E), indicating a reduction in acidity and an improvement in palatability. In a study on soybean paste fermentation, So Woo et al. observed that inoculation with the *Aspergillus oryzae* strain JI4 significantly inhibited OTA production while enhancing the product’s nutritional and sensory quality [50]. These findings highlight the potential of microorganism-fermented foods as a promising strategy for improving food safety and quality.

## 3. Discussion

The biodegradation of OTA has become a prominent research focus among various degradation methods. However, the application of mycotoxin-degrading microorganisms that lack GRAS (Generally Recognized as Safe) status in food and feed systems remains strictly restricted [18]. In this context, probiotic microorganisms—particularly those from the genera *Lactobacillus*, *Bifidobacterium*, and *Weissella*—offer a safe and effective alternative. These probiotics not only exhibit OTA detoxification capacity but also reinforce gut barrier function, thereby providing dual benefits in food safety and host health [51].

Probiotics detoxify OTA mainly via adsorption and enzymatic degradation. The cell walls of *lactic acid bacteria* form OTA-cell complexes through physical adsorption, thereby reducing the bioactivity of OTA [52]. However, adsorption-based detoxification is reversible and may allow OTA desorption [53], resulting in unstable efficiency [54], whereas enzymatic detoxification via strain-secreted hydrolases is generally more thorough and stable. This distinction between reversible adsorption and irreversible enzymatic transformation has been widely emphasized, with enzymatic approaches considered more reliable due to the formation of structurally modified, less toxic metabolites [55]. However, OTA stability is influenced by pH, and this factor should be considered when interpreting degradation results. Previous studies have shown that significant OTA reduction was only observed under strongly alkaline conditions (pH 10) combined with high-temperature treatment (100 °C), whereas under neutral and acidic conditions at 100 °C, no significant OTA reduction occurred [56]. In the present study, our experimental measurements confirmed that OTA remained stable under the experimental conditions (pH 3–9, 37 °C) (Supplementary Figure S2). In this study, *W. coagulans* CGMCC 9951 maintained robust OTA-degrading activity across a wide range of temperatures, pH values, and high OTA concentrations. Activity localization assays revealed that the degradation was primarily driven by extracellular proteases, a mechanism consistent with that reported for *Bacillus subtilis* CW14 [57]. This finding suggests that the extracellular degradation pathway may be particularly advantageous for large-scale applications. Notably, the high OTA degradation activity observed in the VC resuspended in PBS buffer does not contradict the enzyme-based degradation mechanism. Enzymatic catalysis relies on pre-existing enzymes accumulated during cell growth, rather than requiring ongoing enzyme synthesis. This is supported by the observation that *Acinetobacter pittii* AP19 harbors both constitutive and inducible OTA-degrading enzymes, with its viable cell suspension achieving complete OTA removal even in nutrient-free buffer [20]. Furthermore, *W. coagulans* is well-characterized for its secretion of extracellular degrading enzymes [58], which accumulate on the cell surface and in the extracellular matrix during cultivation and retain catalytic activity upon resuspension in PBS.

OTA is degraded through multiple pathways. Proteases, such as carboxypeptidase A, carboxypeptidase PJ-1540, and protease A, catalyze the hydrolysis of the amide bond in OTA, yielding L-β-phenylalanine and the non-toxic metabolite OTα, this pathway is the best-characterized mechanism of OTA degradation [42]. Previous studies have also highlighted that enzymatic hydrolysis of the amide bond is the most well-established detoxification route, consistently leading to OTα formation with markedly reduced toxicity [55]. In addition, cleavage of the C-14 nitrogen atom and the amine group leads to the formation of 3-phenylpropionic acid and OTα-amide, both of which exhibit reduced toxicity [27]. A third pathway involves ochratoxin-lactonase-mediated hydrolysis of the lactone ring, producing open-ring OTA, whose toxicity remains largely unchanged [59]. In this study, MS/MS fragmentation analysis suggests that the major degradation product is consistent with OTα, which has been reported as non-toxic [15]. Animal experiments further demonstrated that OTα did not induce oxidative damage in renal cells of juvenile mice [25]. Moreover, toxicity assessment models showed no significant effects of OTA on hepatocyte viability or teratogenicity in zebrafish embryos [44]. These findings underscore the favorable safety profile of this degradation pathway. Phenylalanine was not detected in our mass spectrometry analysis, yet as an essential amino acid it may be consumed in biological processes; moreover, although degradation products were assigned based on high-resolution MS accurate mass data, verification with authentic OTα standards is needed for definitive structural confirmation.

To date, several OTA-detoxifying enzymes, including carboxypeptidases, amidohydrolases, amidases, lipases, protease A, and Nudix hydrolases, have been reported and functionally characterized [21]. However, only a limited number of these enzymes have been fully purified and mechanistically characterized, highlighting the need for deeper enzymatic and structural studies [55]. Among them, carboxypeptidases are the earliest and most widely investigated and have been identified in microorganisms such as *Acinetobacter pittii* [20], *Lysobacter* sp. [60], and *Brevundimonas naejangsanensis* [61]. In *W. coagulans* CGMCC 9951, a carboxypeptidase (WGU28473.1) with OTA-degrading activity was confirmed via heterologous expression in *E. coli*. However, recent findings indicate that certain amidohydrolases, such as ADH3, exhibit substantially higher catalytic efficiency than conventional carboxypeptidases [41]. This raises the possibility that additional, yet-unidentified enzymes may contribute to the high detoxification efficiency observed in *W. coagulans* CGMCC 9951. While proteomics analysis could provide comprehensive insights into the complete enzymatic network involved in OTA degradation, our current approach successfully identified and validated the key carboxypeptidase responsible for the observed detoxification activity. Future proteomics studies would certainly complement our findings and could reveal additional enzymes that contribute to the remarkable efficiency of this strain. Notably, intracellular fractions of this strain also displayed OTA-degrading activity (Figure 4), mirroring the multi-enzyme synergy reported in *Brevibacillus* sp. ALJ02 and *Cellulomonas* sp. CO29 [18,62]. In *B. naejangsanensis* ML17, four types of enzymes are capable of degrading OTA, however, their combined effects remain unclear [61]. The OTA-degrading enzymes include multiple classes, thus increasing the chances of finding effective detoxifying agents under varying conditions and enhancing the applicability of these microorganisms in practical settings. Understanding the interactions between these enzymes could bring about more effective strategies for OTA degradation.

The irreversibility and non-polluting nature of enzymatic OTA degradation are essential for practical applications [63]. This study represents the first application of *W. coagulans* CGMCC 9951 in the fermentation of raw *Cornus officinalis* pulp. This marks a novel approach to utilizing *W. coagulans* CGMCC 9951 in food processing, demonstrating its potential for both mycotoxin detoxification and food safety enhancement. The *Cornus officinalis* pulp, rich in sugars and organic acids, not only provides sufficient nutrients to support cell growth and continuous extracellular enzyme secretion by *W. coagulans* CGMCC 9951 but also offers a practical and economically viable strategy for food applications. Similarly, *Oenococcus oeni* has been reported to maintain system stability and improve sensory properties during grape juice fermentation [64], and both this strain and *cocci* isolated from grape juice exhibit OTA-degrading capability [28]. Such examples illustrate the broader potential of fermentation microbiota to mitigate mycotoxin risks while optimizing product characteristics.

Overall, *W. coagulans* CGMCC 9951 exhibits a robust OTA detoxification system that may converts OTA into the non-toxic metabolite OTα. Its successful application in food fermentation highlights its potential as a safe and effective biocontrol agent. This study not only advances the understanding of probiotic-mediated mycotoxin degradation but also provides a practical framework for developing GRAS-compliant detoxification strategies in food systems.

## 4. Conclusions

This study identified *W. coagulans* CGMCC 9951 as a highly efficient OTA-degrading probiotic, achieving 98% degradation of OTA at 300 ng/mL within 72 h. Notably, degradation by this strain was mediated by heat-sensitive, metal-dependent extracellular enzymes sensitive to proteinase K, which likely cleave the amide bond in OTA, and produce the non-toxic metabolite OTα. We also expressed and purified the carboxypeptidase (WGU28473.1) in *E. coli*, and the purified enzyme reduced OTA levels by 46%. Molecular docking also revealed strong interactions between WGU28473.1 and OTA (binding energy: −8.4 kcal/mol), suggesting that this enzyme plays a role in the degradation process of strain *W. coagulans* CGMCC 9951. *W. coagulans* CGMCC 9951 not only significantly reduced OTA levels in *Cornus officinalis* pulp but also improved food safety and sensory quality by reducing astringency and enhancing palatability. Collectively, these findings provide a theoretical basis for developing probiotic-based detoxification strategies for the food and feed industries.

## 5. Materials and Methods

### 5.1. Reagents and Materials

OTA (purity ≥ 98%, CAS 303-47-9, Romer, Shanghai Baishun Biotechnology Co., Ltd., Shanghai, China). Methanol, acetonitrile, glacial acetic acid (HPLC grade), chloroform, and proteinase K solution (20 mg/mL) were sourced from Beijing LanJeiKe Technology Co., Ltd., Beijing, China. Phenol, glucose, DPPH (2,2-diphenyl-1-picrylhydrazyl), 95% ethanol, ABTS (2,2′-azino-bis (3-ethylbenzothiazoline-6-sulfonic acid)), potassium persulfate, and other analytical grade chemicals required no further purification. *Cornus officinalis* pulp was prepared by the NFC (non-concentrated reduced juice) process for aseptic canning.

The PBS buffer contained 8 g/L NaCl, 0.2 g/L KCl, 3.63 g/L Na_2_HPO_4_·12H_2_O, and 0.24 g/L KH_2_PO_4_. The liquid medium (NJ) contained 15 g/L glucose, 15 g/L tryptone, 10 g/L yeast powder, and 5 g/L MgSO_4_. Lysogeny Broth (LB) contained 10 g/L tryptone, 10 g/L NaCl, and 5 g/L yeast extract (kanamycin was added to a final concentration of 50 μg/mL after sterilization). All media were sterilized at 115 °C for 30 min. Subsequent to medium sterilization, OTA was added to achieve a final concentration of 300 ng/mL (OTA contaminated NJ). The NJ solid medium contained 20 g/L of agar.

### 5.2. Strain Culture

The *W. coagulans* CGMCC 9951 is maintained at the School of Food and Biological Engineering, Henan University of Science and Technology. Colonies were isolated on NJ solid medium, then transferred to liquid medium and shaken at 37 °C for 24 h. The bacterial suspension used for inoculation had an approximate concentration of 8 × 10^7^ CFU/mL, as determined by standard plate counting. The inoculum concentration was kept consistent across all experiments to ensure comparability of results.

*Escherichia coli* Trans1-T1 (SYNBIO Technologies, Suzhou, China) was used for general cloning, while *E. coli* BL21 (DE3) (SYNBIO Technologies, Suzhou, China) was utilized for protein expression. The plasmid pET-28a (+), which carries a kanamycin resistance gene and was obtained from SYNBIO Technologies (Suzhou, China), served as the expression vector. All *E. coli* strains were cultured in LB at 37 °C.

### 5.3. UPLC Quantification of OTA and Chromatographic Conditions

A 2% inoculum of *W. coagulans* CGMCC 9951 was added to NJ medium and cultured for 24 h. Samples were collected every 2 h, and the absorbance at 600 nm (OD_600_) was measured with three biological replicates. The growth curve was plotted with time as the abscissa and absorbance as the ordinate.

*W. coagulans* CGMCC 9951 was inoculated into OTA contaminated NJ medium and shaken at 180 rpm at 37 °C. Samples were collected at 4 h, 8 h, 12 h, 24 h, 48 h, 72 h, 96 h, and 120 h. NJ medium without OTA was the control, with three replicates per group.

OTA was extracted from samples using chloroform and quantified by UPLC. Determination was performed using reverse-phase UHPLC (Waters, Milford, MA, USA), with a fluorescence detector and an XB-C18 reverse-phase column (2.1 mm × 100 mm, 1.8 µm). The excitation and emission wavelengths for fluorescence detection were 310 nm and 465 nm, respectively. The mobile phase (acetonitrile: water: acetic acid, 48:51:1) was pumped at 0.3 mL/min. Sample injection volume was 2 µL, column temperature was 35 °C.

The OTA degradation rate was calculated with Formula (1):

OTA degradation rate (%) = (1 − residual OTA peak area post-degradation/initial OTA peak area) × 100%(1)

### 5.4. Effect of Fermentation Factors on the Degradation of OTA by W. coagulans CGMCC 9951

The OTA degradation rate was assessed under varying conditions, including temperature, inoculation volume, rotation speed, and pH. Temperature tests were conducted at 180 rpm and pH 7.0, using 17 °C, 27 °C, 37 °C, 47 °C, and 57 °C. The effects of inoculation volume were measured at 37 °C. The corresponding initial microbial loads for inoculation volumes of 2%, 4%, 6%, 8%, and 10% were approximately 1.53 × 10^5^ CFU/mL, 2.52 × 10^5^ CFU/mL, 5 × 10^5^ CFU/mL, 5.7 × 10^5^ CFU/mL, and 6.4 × 10^5^ CFU/mL respectively. At 37 °C with 2% inoculation, rotation speeds (140 rpm, 160 rpm, 180 rpm, 200 rpm, and 220 rpm) were tested. The impact of pH was evaluated at pH 3.0, 4.0, 5.0, 6.0, 7.0, 8.0, and 9.0. The pH of each NJ medium sample was adjusted to the desired value using HCl or NaOH prior to inoculation. To evaluate the abiotic effect of pH on OTA stability, uninoculated controls containing NJ medium with OTA (300 ng/mL) at each pH value (3.0–9.0) were incubated under the same conditions (37 °C, 180 rpm, 24 h). All samples contained OTA at 300 ng/mL and were incubated for 24 h. The control was NJ incubated with OTA under the same conditions. All samples were analyzed according to Formula (1) (Section 5.3).

### 5.5. Influence of Initial OTA Concentration on the Growth and Degradation Rate of W. coagulans CGMCC 9951

A 2% inoculum of *W. coagulans* CGMCC 9951 was added to the culture medium, and OTA concentration gradients were established at 300 ng/mL, 1000 ng/mL, 3000 ng/mL and 5000 ng/mL (*n* = 3). Samples were collected at 12 h, 24 h, 48 h, 72 h, 96 h, and 120 h, and analyzed according to Formula (1) (Section 5.3). The bacterial growth was determined by measuring OD_600_.

### 5.6. Analysis of Active Fraction Contributing to OTA Degradation by W. coagulans CGMCC 9951

The degradation of OTA by various cellular components of *W. coagulans* CGMCC 9951 was systematically investigated. A 12 h culture of *W. coagulans* CGMCC 9951 was subjected to centrifugation at 12,000 rpm for 10 min at 4 °C. The resulting culture supernatant was filtered with a 0.22 µm syringe filter to yield the cell-free supernatant (CF). The cell pellets were washed twice and then resuspended in phosphate-buffered saline (PBS, pH 7.2–7.4) to prepare the viable cell suspension (VC). The VC was disrupted on ice using an ultrasonic cell disruptor for 30 min, then centrifuged at 12,000 rpm and 4 °C for 10 min to obtain the cell lysate (CL). The VC was also autoclaved at 115 °C for 30 min to produce the heat-inactivated cell suspension (HI). For OTA degradation assays, 994 µL of CF, VC, CL, and HI were combined with 6 µL of a 50 µg/mL OTA solution, respectively. The reaction mixtures were incubated at 37 °C for 12 h, 24 h, 48 h, and 72 h. Each experiment was conducted in triplicate. The blank control group for CF consisted of sterile NJ medium, while for the groups of VC, CL, and HI, sterile PBS (pH 7.2–7.4) was used as the control. Results were analyzed according to the specified analytical method (Formula (1) (Section 5.3)).

### 5.7. The Stability of W. coagulans CGMCC 9951 Fermentation Supernatant for OTA Degradation

For the heat treatment test, the cell-free supernatant (CF) was heated to 100 °C for 30 min. For the anionic surfactant treatment, CF was treated with a 50 mg/mL solution of sodium dodecyl sulfate (SDS) at 37 °C for 1 h. For the protease K treatment test, CF was treated with protease K at a final concentration of 1 mg/mL for 1 h at 55 °C or incubated at 55 °C without protease K for 1 h. The CF without treatment served as the control. OTA was then added to all treatments at a final concentration of 300 ng/mL, followed by further incubation at 37 °C and 180 rpm for 12 h.

In the metal-chelator treatment test, 900 µL of CF was mixed with EDTA (1 M, dissolved in PBS, pH 7.2–7.4) to a final volume of 1 mL, and the mixture was incubated at 37 °C for 1 h. The control included the same amount of buffer without EDTA. The final EDTA concentrations were 0.1 mM, 0.5 mM, 1 mM, and 10 mM. OTA was then added to a final concentration of 300 ng/mL, followed by further incubation at 37 °C and 180 rpm for 12 h.

For metal ion treatment, 900 µL CF and 100 µL each of metal ion solution (100 mM in PBS, pH 7.2–7.4) and incubated at 37 °C for 1 h. The metal ion solutions were prepared by adding NaCl, CuSO_4_, CaCl_2_, K_2_SO_4_, FeCl_3_, LiOH, FeSO_4_, MnSO_4_, BaCl_2_, and MgSO_4_ to PBS (pH 7.2–7.4). A mixture of 900 µL CF and 100 µL buffer was used as a control. After incubation, OTA was added to a final concentration of 300 ng/mL, followed by further incubation at 37 °C and 180 rpm for 12 h.

For organic solvent treatment, 900 µL CF, 10 µL or 50 µL organic solvent, and PBS (pH 7.2–7.4) were combined to 1 mL and incubated at 37 °C for 1 h. The solvents tested included Tween 80, acetone, ethanol, and methanol. OTA was then added to a final concentration of 300 ng/mL. The final concentrations of organic solvents were 1% or 5%. After OTA addition, the mixtures were further incubated at 37 °C and 180 rpm for 12 h.

After incubating the mixtures at 37 °C and 180 rpm for 12 h, the residual OTA levels were measured to calculate the OTA degradation rate.

### 5.8. UHPLC-MS Analysis

The OTA stock solution was added to the bacterial culture for a final concentration of 300 ng/mL. The culture was then incubated at 37 °C, shaken at 180 rpm for 72 h. Afterwards, 1 mL of culture was taken, centrifuged, and the supernatant was filtered through a 0.22 µm membrane to obtain the test sample.

Detection was performed using a Vanquish Flex ultra-high-performance liquid chromatography (UHPLC) system coupled with a Thermo Scientific Orbitrap Exploris 120 mass spectrometer (MS) at Shiyanjia Lab (Hangzhou, China). The samples were injected onto an ACQUITY BEH C18 column (100 mm × 2.1 mm, 1.7 µm) with the column maintained at 40 °C. The injection volume was 2 µL and the flow rate was 0.3 mL/min. Mobile phase A was a 0.1% formic acid aqueous solution, while mobile phase B was acetonitrile. Mass spectrometry was performed under the following conditions: ion source: heated electrospray ionization (HESI); sheath gas rate: 40 mL/min; auxiliary gas rate: 8 mL/min; spray voltage: 2.8 kV in negative ion mode; capillary temperature: 320 °C; S-lens: 50%; auxiliary gas temperature: 300 °C. The scan mode used was full mass spectrometry (Full MS), with a scan range of 100–1500 *m*/*z*. For structural confirmation of the degradation product, MS/MS analysis was additionally performed using collision-induced dissociation, with product ions acquired starting at *m*/*z* 50.

### 5.9. Expression and Activity Assay of Recombinant WGU28473.1

The gene sequence of WGU28473.1 was retrieved from the genome of *W. coagulans* CGMCC 9951 (locus tag: MKO99_00365) and codon-optimized for heterologous expression in *E. coli*. The codon-optimized gene was chemically synthesized and directionally cloned into the pET-28a (+) vector using BamH I and Xho I restriction sites by Suzhou Hongxun Biotechnology Co., Ltd. (Suzhou, China). The recombinant plasmid was introduced into *E. coli* BL21 (DE3) for overexpression. After culturing and induction, the cells were lysed, and the crude enzyme was collected by centrifugation at 12,000× *g* for 20 min. Protein expression was analyzed by sodium dodecyl sulfate–polyacrylamide gel electrophoresis (SDS-PAGE). OTA degradation was determined by incubating 0.5 mL crude enzyme with 0.5 mL OTA standard solution (300 ng/mL, prepared in PBS) at 37 °C for 1 h. The crude enzyme was further purified using a His-tag Protein Purification Kit (Lot No. A438251016, Beyotime Biotechnology, Shanghai, China), concentrated and buffer-exchanged using Cobetter 10 kDa Briscale™ UF centrifugal filters (Hangzhou Cobetter Filtration Equipment Co., Ltd., Hangzhou, China), and the protein concentration was determined using a BCA Protein Assay Kit (Beijing Biosharp, Beijing, China).

### 5.10. Molecular Docking

The 3D structure of WGU28473.1 was obtained by homology modeling using SWISS-MODEL based on its amino acid sequence, and subsequently optimized using Discovery Studio 2019 (water removal, hydrogen addition, charge and side chain completion). The ligand structure was downloaded from PubChem and energy-minimized in Discovery Studio 2019. Both protein and ligand were converted to PDBQT format using AutoDock Tools (Version 1.5.6). A grid box covering the entire protein was set to define the binding search space. Docking was performed using AutoDock Vina (Version 1.2.6). Results were visualized with PyMOL (Version 3.1).

### 5.11. Application Exploration of W. coagulans CGMCC 9951 for OTA Degradation in Cornus officinalis Pulp

The fermentation broth of *W. coagulans* CGMCC 9951 were harvest at the logarithmic growth phase, then resuspend the cells in 0.9% saline solution. *Cornus officinalis* pulp was diluted with sterile, purified water to serve as the fermentation substrate. Four groups were established: Group 1 served as the control group. Group 2 was supplemented with 2% VC, designated as the YS group. Group 3 was supplemented with 2% VC and OTA to a final concentration of 300 ng/mL, referred to as the YSO group. Group 4 was supplemented with OTA to a final concentration of 300 ng/mL, designated as the SO group. All groups were fermented at 37 °C, 180 rpm. The third group samples were collected every 24 h to measure OTA levels. Fermentation was terminated when OTA levels achieved stability.

The sugar content, antioxidant activity, pH value, and total acidity of each group were tested. The glucose concentration was measured via a standard curve, adapted from Zhang et al. [65], with measurements conducted under identical conditions. The DPPH and ABTS^+^ free radical scavenging activities were determined according to Zhang et al. [66]. The pH and total acid content were measured according to GB 12456-2021 [67].

### 5.12. Statistical Analysis

All statistical analyses were completed using SPSS 20.0 (SPSS, IBM, Chicago, IL, USA) software. One-way analysis of variance (ANOVA) was performed to determine statistically significant differences (*p* < 0.05). Mean values from three replicates are presented as mean ± standard deviation (SD).

## Data Availability

The original contributions presented in this study are included in the article. Further inquiries can be directed to the corresponding author.

## Supplementary Materials

The following supporting information can be downloaded at: https://www.mdpi.com/article/10.3390/toxins18050194/s1. Table S1. The DNA sequence of the recombinant fusion protein; Figure S1. Growth curve of *W. coagulans* CGMCC 9951 (Five days); Figure S2. Relative content of OTA in media with different pH values; Figure S3. MS/MS spectrum of the OTA degradation product; Figure S4. SDS-PAGE analysis of WGU28473.1; Figure S5. OTA degradation activity of purified WGU28473.1.

## References

[1] Lan W., Xia H., Jie S., Ninghao J., Ting J., Bing L., Lijun L., Yun C. (2022). Ochratoxin A: Occurrence and recent advances in detoxification. Toxicon.

[2] Hassan R., Gonzalez D., Hobloss Z., Brackhagen L., Myllys M., Friebel A., Seddek A.L., Marchan R., Cramer B., Humpf H.U. (2022). Inhibition of cytochrome P450 enhances the nephro- and hepatotoxicity of ochratoxin A. Arch. Toxicol..

[3] Frangiamone M., Lozano M., Cimbalo A., Font G., Manyes L. (2023). AFB1 and OTA Promote Immune Toxicity in Human LymphoBlastic T Cells at Transcriptomic Level. Foods.

[4] Stoev S.D. (2013). Food safety and increasing hazard of mycotoxin occurrence in foods and feeds. Crit. Rev. Food Sci. Nutr..

[5] Marin-Kuan M., Nestler S., Verguet C., Bezencon C., Piguet D., Mansourian R., Holzwarth J., Grigorov M., Delatour T., Mantle P. (2006). A toxicogenomics approach to identify new plausible epigenetic mechanisms of ochratoxin a carcinogenicity in rat. Toxicol. Sci..

[6] Shi H.T., Li S.L., Bai Y.Y., Prates L.L., Lei Y.G., Yu P.Q. (2018). Mycotoxin contamination of food and feed in China: Occurrence, detection techniques, toxicological effects and advances in mitigation technologies. Food Control.

[7] Sharma A. (2018). Evaluation of certain food additives and contaminants. Eightieth report of the Joint FAO/WHO Expert Committee on Food Additives. World Health Organization technical report series. Indian J. Med. Res..

[8] Ostry V., Malir F., Toman J., Grosse Y. (2017). Mycotoxins as human carcinogens-the IARC Monographs classification. Mycotoxin Res..

[9] Freire L., Braga P.A.C., Furtado M.M., Delafiori J., Dias-Audibert F.L., Pereira G.E., Reyes F.G., Catharino R.R., Sant’Ana A.S. (2020). From grape to wine: Fate of ochratoxin A during red, rose, and white winemaking process and the presence of ochratoxin derivatives in the final products. Food Control.

[10] Ałtyn I., Twarużek M. (2020). Mycotoxin Contamination Concerns of Herbs and Medicinal Plants. Toxins.

[11] Qin L., Jiang J.Y., Zhang L., Dou X.W., Ouyang Z., Wan L., Yang M.H. (2020). Occurrence and analysis of mycotoxins in domestic Chinese herbal medicines. Mycology.

[12] Wang L., Su D., Yuan Q., Xiao C., Hu M., Guo L., Kang C., Zhang J., Zhou T. (2024). Simultaneous detection of multiple mycotoxins in Radix Dipsaci and estimation of exposure risk for consumers. Sci. Rep..

[13] Elamin A., Sakuda S. (2025). Mechanism of Mycotoxin Contamination of Medicinal Herbs. Toxins.

[14] Deng W., Liu Y., Guo Y., Chen J., Abdu H.I., Khan M.R.U., Palanisamy C.P., Pei J., Aty A.M.A.E. (2024). A comprehensive review of *Cornus officinalis*: Health benefits, phytochemistry, and pharmacological effects for functional drug and food development. Front. Nutr..

[15] Zhang L., Zhang X., Chen X., Zhang W., Zhao L., Wang Z., Guo Y. (2024). Biodegradation of ochratoxin A by *Brevundimonas diminuta* HAU429: Characterized performance, toxicity evaluation and functional enzymes. Food Res. Int..

[16] Nobre C., Gonzalez A., Losoya C., Teixeira J.A., Belmares R., Abrunhosa L. (2022). Detoxification of ochratoxin A and zearalenone by Pleurotus ostreatus during in vitro gastrointestinal digestion. Food Chem..

[17] Wang L., Liu X., Cai R., Ge Q., Zhao Z., Yue T., Yuan Y., Gao Z., Wang Z. (2022). Detoxification of Ochratoxin A by pulsed light in grape juice and evaluation of its degradation products and safety. Innov. Food Sci. Emerg. Technol..

[18] Yang Y., Zhong W.T., Liu Z.H., Xue X.L., Gao Q., Wang D.P., Zhang Y., Zhang J. (2023). Isolation and identification of a *Cytobacillus oceanisediminis* strain with ochratoxin A detoxification ability. Food Control.

[19] Sun Z., You Y.X., Xu H.D., You Y., He W.J., Wang Z.P., Li A.T., Xia Y. (2024). Food-Grade Expression of Two Laccases in *Pichia pastoris* and Study on Their Enzymatic Degradation Characteristics for Mycotoxins. J. Agric. Food Chem..

[20] Yang Y., Zhong W., Wang Y., Yue Z., Zhang C., Sun M., Wang Z., Xue X., Gao Q., Wang D. (2024). Isolation, identification, degradation mechanism and exploration of active enzymes in the ochratoxin A degrading strain *Acinetobacter pittii* AP19. J. Hazard. Mater..

[21] Fu X., Fei Q., Zhang X., Li N., Zhang L., Zhou Y. (2024). Two different types of hydrolases co-degrade ochratoxin A in a highly efficient degradation strain *Lysobacter* sp. CW239. J. Hazard. Mater..

[22] Zhao Z., Niu Z., Liang Z. (2024). Ochratoxin A Degradation and Stress Response Mechanism of *Brevundimonas naejangsanensis* ML17 Determined by Transcriptomic Analysis. Foods.

[23] Chen N., Fei Q., Luo H., Fang Z., Xiao Y., Du Z., Zhou Y. (2022). Isoenzyme N-Acyl-l-Amino Acid Amidohydrolase NA Increases Ochratoxin A Degradation Efficacy of *Stenotrophomonas* sp. CW117 by Enhancing Amidohydrolase ADH3 Stability. Microbiol. Spectr..

[24] Liu S., He Y., He W., Song X., Peng Y., Hu X., Bian S., Li Y., Nie S., Yin J. (2024). Exploring the Biogenic Transformation Mechanism of Polyphenols by *Lactobacillus plantarum* NCU137 Fermentation and Its Enhancement of Antioxidant Properties in Wolfberry Juice. J. Agric. Food Chem..

[25] Xiong K., Wang X.L., Zhi H.W., Sun B.G., Li X.T. (2017). Identification and safety evaluation of a product from the biodegradation of ochratoxin A by an *Aspergillus* strain. J. Sci. Food Agric..

[26] Gu K.J., Ryu D., Lee H.J. (2021). Ochratoxin A and its reaction products affected by sugars during heat processing. Food Chem..

[27] Bittner A., Cramer B., Harrer H., Humpf H.U. (2015). Structure elucidation and in vitro cytotoxicity of ochratoxin α amide, a new degradation product of ochratoxin A. Mycotoxin Res..

[28] Abrunhosa L., Ines A., Rodrigues A.I., Guimaraes A., Pereira V.L., Parpot P., Mendes-Faia A., Venancio A. (2014). Biodegradation of ochratoxin A by *Pediococcus parvulus* isolated from Douro wines. Int. J. Food Microbiol..

[29] Sanchez-Arroyo A., Plaza-Vinuesa L., Mancheno J.M., de Las Rivas B., Munoz R. (2025). Brevibacterium enzymes as biological tools for ochratoxin A detoxification in dairy foods. Int. J. Food Microbiol..

[30] Rodriguez H., Reveron I., Doria F., Costantini A., De las Rivas B., Munoz R., Garcia-Moruno E. (2011). Degradation of ochratoxin A by *Brevibacterium* species. J. Agric. Food Chem..

[31] Ondiek W., Wang Y.L., Sun L.J., Zhou L.H., On S.L.W., Zheng H.T., Ravi G. (2022). Removal of aflatoxin b1 and t-2 toxin by bacteria isolated from commercially available probiotic dairy foods. Food Sci. Technol. Int..

[32] Piotrowska M. (2014). The Adsorption of Ochratoxin A by *Lactobacillus* Species. Toxins.

[33] Mozaffary P., Milani J.M., Heshmati A. (2019). The influence of yeast level and fermentation temperature on Ochratoxin A decrement during bread making. Food Sci. Nutr..

[34] Cho S.M., Jeong S.E., Lee K.R., Sudhani H.P., Kim M., Hong S.Y., Chung S.H. (2016). Biodegradation of Ochratoxin A by *Aspergillus tubingensis* Isolated from Meju. J. Microbiol. Biotechnol..

[35] Ismaiel A.A., Mohamed H.H., El-Sayed M.T. (2022). Biodegradation of ochratoxin A by endophytic *Trichoderma koningii* strains. World J. Microbiol. Biotechnol..

[36] Badji T., Durand N., Bendali F., Piro-Metayer I., Zinedine A., Ben Salah-Abbès J., Abbès S., Montet D., Riba A., Brabet C. (2023). In vitro detoxification of aflatoxin B1 and ochratoxin A by lactic acid bacteria isolated from Algerian fermented foods. Biol. Control.

[37] Ábrahám R., Baka E., Nussairawi M.A., Táncsics A., Farkas M., Nagy I., Kriszt B., Cserháti M. (2025). Molecular insights into ochratoxin A biodegradation. Biol. Futur..

[38] Liuzzi V.C., Francesca F., Mariana T., Miriam H., Ernesto P., Caterina M., Claudia L., Francesco G., Logrieco A.F., Thon M.R. (2017). Transcriptional Analysis of *Acinetobacter* sp. neg1 Capable of Degrading Ochratoxin A. Front. Microbiol..

[39] Qian Y., Zhang X., Fei Q., Zhou Y. (2021). Comments on the ochratoxin A degradation mechanism by *Lysobacter* sp. CW239—Wei Wei et al. (2020). Environ. Pollut..

[40] Sanchez-Arroyo A., Plaza-Vinuesa L., de Las Rivas B., Mancheno J.M., Munoz R. (2024). *Aspergillus niger* Ochratoxinase Is a Highly Specific, MetalDependent Amidohydrolase Suitable for OTA Biodetoxification in Food and Feed. J. Agric. Food Chem..

[41] Luo H., Wang G., Chen N., Fang Z.M., Xiao Y.Z., Zhang M., Gerelt K., Qian Y.Y., Lai R., Zhou Y. (2022). A Superefficient Ochratoxin A Hydrolase with Promising Potential for Industrial Applications. Appl. Environ. Microbiol..

[42] Jia R., Zhao J., Tian S., Sadiq F.A., Lu S., Gao P., Zhang G. (2025). Enzymatic degradation of Ochratoxin A by a novel bacterium, *Microbacterium esteraromaticum* ASAG1016. Int. J. Food Microbiol..

[43] Zhang X., Li J., Cheng Z., Zhou Z., Ma L. (2016). High-performance liquid chromatography-tandem mass spectrometry method for simultaneous detection of ochratoxin A and relative metabolites in *Aspergillus* species and dried vine fruits. Food Addit. Contam. Part A Chem. Anal. Control Expo. Risk Assess..

[44] Haq M., Gonzalez N., Mintz K., Jaja-Chimedza A., De Jesus C.L., Lydon C., Welch A., Berry J.P. (2016). Teratogenicity of Ochratoxin A and the Degradation Product, Ochratoxin alpha, in the Zebrafish (*Danio rerio*) Embryo Model of Vertebrate Development. Toxins.

[45] Teufel R., Mascaraque V., Ismail W., Voss M., Perera J., Eisenreich W., Haehnel W., Fuchs G. (2010). Bacterial phenylalanine and phenylacetate catabolic pathway revealed. Proc. Natl. Acad. Sci. USA.

[46] Abrunhosa L., Santos L., Venâncio A. (2006). Degradation of Ochratoxin A by Proteases and by a Crude Enzyme of *Aspergillus niger*. Food Biotechnol..

[47] Chang X.J., Wu Z.D., Wu S.L., Dai Y.S., Sun C.P. (2015). Degradation of ochratoxin A by *Bacillus amyloliquefaciens* ASAG1. Food Addit. Contam A.

[48] Bontsidis C., Mallouchos A., Terpou A., Nikolaou A., Batra G., Mantzourani I., Alexopoulos A., Plessas S. (2021). Microbiological and Chemical Properties of Chokeberry Juice Fermented by Novel Lactic Acid Bacteria with Potential Probiotic Properties during Fermentation at 4 degrees C for 4 Weeks. Foods.

[49] Andres C.M.C., Perez de la Lastra J.M., Juan C.A., Plou F.J., Perez-Lebena E. (2024). Antioxidant Metabolism Pathways in Vitamins, Polyphenols, and Selenium: Parallels and Divergences. Int. J. Mol. Sci..

[50] Woo S.Y., Tian F., Lee S.Y., Park S.B., Han K.-H., Kim H.-Y., Chun H.S. (2024). Reduction of aflatoxins and ochratoxin A by addition of commercial Koji during fermentation of the Korean traditional soybean paste, Doenjang. Food Control.

[51] Sánchez-Arroyo A., Plaza-Vinuesa L., de las Rivas B., Mancheño J.M., Muñoz R. (2025). Unravelling OTA-detoxification by the dioxin-mineralizing bacterium *Rhizorhabdus wittichii* RW1T. Int. Biodeterior. Biodegrad..

[52] Mwabulili F., Xie Y.L., Li Q., Sun S.M., Yang Y.H., Ma W.B. (2022). Research progress of ochratoxin a bio-detoxification. Toxicon.

[53] Piotrowska M., Zakowska Z. (2005). The elimination of ochratoxin A by lactic acid bacteria strains. Pol. J. Microbiol..

[54] Peteri Z., Teren J., Vagvolgyi C., Varga J. (2007). Ochratoxin degradation and adsorption caused by astaxanthin-producing yeasts. Food Microbiol..

[55] Loi M., Fanelli F., Liuzzi V., Logrieco A., Mulè G. (2017). Mycotoxin Biotransformation by Native and Commercial Enzymes: Present and Future Perspectives. Toxins.

[56] Samjhana D., Jung L.H., Kejia G., Dojin R. (2016). Heat Stability of Ochratoxin A in an Aqueous Buffered Model System. J. Food Prot..

[57] Hu H.N., Jia X., Wang Y.P., Liang Z.H. (2018). Removal of ochratoxin A by a carboxypeptidase and peptides present in liquid cultures of CW14. World Mycotoxin J..

[58] Maresca E., Aulitto M., Contursi P. (2024). Harnessing the dual nature of *Bacillus* (Weizmannia) *coagulans* for sustainable production of biomaterials and development of functional food. Microb. Biotechnol..

[59] Leitao A.L., Enguita F.J. (2021). Systematic structure-based search for ochratoxindegrading enzymes in proteomes from filamentous fungi. Biomolecules.

[60] Wei W., Qian Y.Y., Wu Y.B., Chen Y., Peng C., Luo M.Z., Xu J.F., Zhou Y. (2020). Detoxification of ochratoxin A by *Lysobacter* sp. CW239 and characteristics of a novel degrading gene carboxypeptidase. Environ. Pollut..

[61] Peng M.X., Zhang Z.Z., Xu X.E., Zhang H.X., Zhao Z.T., Liang Z.H. (2023). Purification and characterization of the enzymes from *Brevundimonas naejangsanensis* that degrade ochratoxin A and B. Food Chem..

[62] Liu C.C., Zhao C.C., Liu H.J., Du W., Sun J., Zhou W.H., Sun C.P. (2023). Biodegradation of ochratoxin A by two novel strains of *Brevibacillus* sp. isolated from wheat (*Triticum aestivum* L.). Food Biosci..

[63] Zhao G.Z., Wang Y.F., Chen J.L., Yao Y.P. (2020). Predominant Mycotoxins, Pathogenesis, Control Measures, and Detection Methods in Fermented Pastes. Toxins.

[64] Mateo E.M., Medina A., Mateo F., Valle-Algarra F.M., Pardo I., Jiménez M. (2010). Ochratoxin A removal in synthetic media by living and heat-inactivated cells of isolated from wines. Food Control.

[65] Zhang W.H., Wu J., Weng L.Y., Zhang H.J., Zhang J., Wu A.B. (2020). An improved phenol-sulfuric acid method for the determination of carbohydrates in the presence of persulfate. Carbohydr. Polym..

[66] Zhang M., Zhang T., Chen C., Yang W., Zhao M., Li Q., Tian J., Zhao Y., Zhang B. (2025). Peptide profiling and antioxidant characterization of the simulated gastrointestinal digest of hemp seed proteins. Food Chem..

[67] (2021). Determination of Total Acid in Foods.

