# Supplementary material for: Detoxification of Ochratoxin a by Weizmannia coagulans CGMCC 9951: Characterization, Mechanism, and Application in Cornus officinalis Pulp
Source: Toxins (Basel). 2026 Apr 22;18(5):194. doi: 10.3390/toxins18050194 (PMC13211256; doi:10.3390/toxins18050194)
Supplement: Supplementary file 1 [file toxins-18-00194-s001.zip › toxins-4232387-supplementary.pdf]

toxins

MDPI

Supplementary Material

Detoxification of Ochratoxin A by *Weizmannia coagulans* CGMCC 9951: Characterization, Mechanism, and Application in *Cornus officinalis* pulp

Table S1. The DNA sequence of the recombinant fusion protein

	DNA sequences
Carboxypeptidase	
WGU28473.1	GTGAAAAAGAGCCTGCAGAAAATGGCGTTT-<br>GCGTTTCTGGCGCTGCTGTTAAC-<br>CGCCGGCGTTGTGCAGCAGGTGCCGAAAACTGTGCATGCGCAGGATCAG<br>CTGGATATTCATGCGAAAGCGGCGATTATTGTGGATGCGGATAAC-<br>GGCCAGATTCTG-<br>TATGAAAAGAACGCGAACCAGGCGCTGGGCATTGCGAGCATGAGCAAA<br>ATGATGACCGAGTACCTGCTGCTGAAAGCGATTCATGA-<br>GAAAAAGGTGAGCTGGAAC-<br>CAGAAAGTGACCATTAGCGATTATGCGTATCGCATTAGCCAGAACCGCG<br>CGCTGAGCAACGTGCCGCTGCGTAAAGGCGAAAAATATACCGTGAAA-<br>GAACTGTTTCAGGCGATGGCGATTTATAGCGCGAACGGCGCGACCATTG<br>CGATTGCAGAAACCCTGGGCGGTACCGAAAAGAAC-<br>TATCTGAACATGATGAACAAACAGGCGAAAGCGTTTGGCCTGACCGATT<br>ATAAATTTGTGAACGCGACCGGCCTGAACAACGAAGATCTGCAGGG-<br>CATGCAGCCG-<br>GAAGGCACTGGCCGCACCGAAGAAAACCTGGTGAGTGCGAAAAGCGTG<br>GCGAAAATTGCGTATCATCTGATTCACGACTATCCGGAGATTCTG-<br>CAGACCACCAGCAC-<br>CGCGAAAATGAAATTTCGCGCGGGCACCGATGATGAAATTGCGATGCAG<br>AACTGGAACTGGATGTTACCGAGCCTGGTTTATGGCCGCCAGGGCGTG-<br>GATGGCTTAAAAAC-<br>CGGCAACACCGATAACGCGGGCTATTGCTTTACCGGCACCGCGAAACAG<br>AACGGCATGCGCATTATTACCGTGGTGCTGCATGCGGAAGATGCGAAC-<br>GGCAACAGCAC-<br>CATTAAAAGCCGCTTTGATGTGACCAACAAACTGATGGATTATGCGTTTA<br>GCAACTTTACCGAAAAGACCCTGTATCCGAAAGGC-<br>TATCAGGTGAAAAAGACCGTGAC-<br>CGTGAAAAGCGGCAAAGAAAAACAGGCGAGCGTGGTGACCAAAGCGC<br>CGCTGAAAGTGGCGGTGAAAAAC-<br>GGCGAAGGCAAAATGTATACCCCGGTGGTGAAATTTGA-<br>TAGCAAACAGATTAAAGCGCCGGTGAAAAAGGGCACCAAAGTGGGCAC

	CCTGTATGCGCGCCATAAAGATGGCCAGGAACTGGGCTATCTG-TATGGCCCGAACACCGCG-CATACCGCGCTGGTGACCAAAACCGATATTGAAAAAGCGAACTGGTTTGTGCGCATGATGCGCGGCATTGGCGGCTTTTTCGGCGGCATTTGGGATCAT-ACCGTGGGCAAACTGTTTTAA

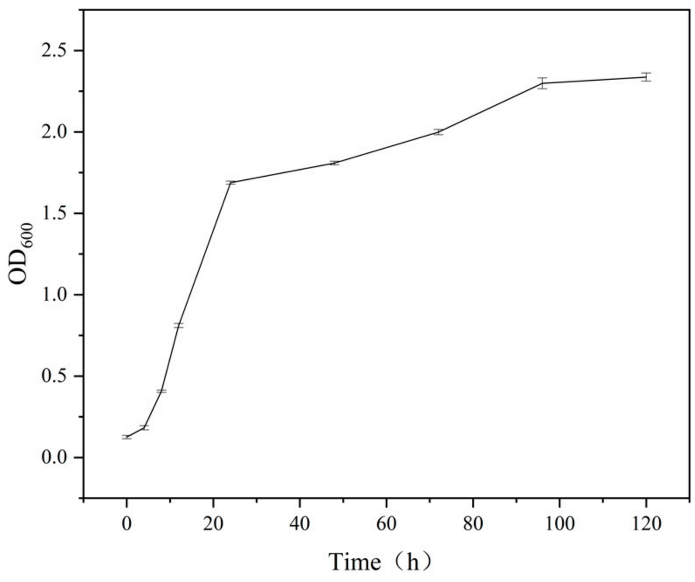

Figure S1. Growth curve of *W. coagulans* CGMCC 9951 (Five days).

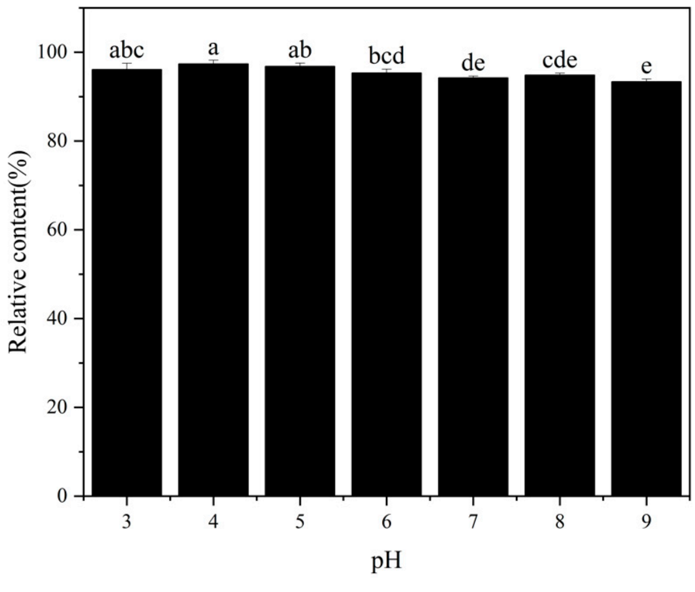

Figure S2. Relative content of OTA in media with different pH values.

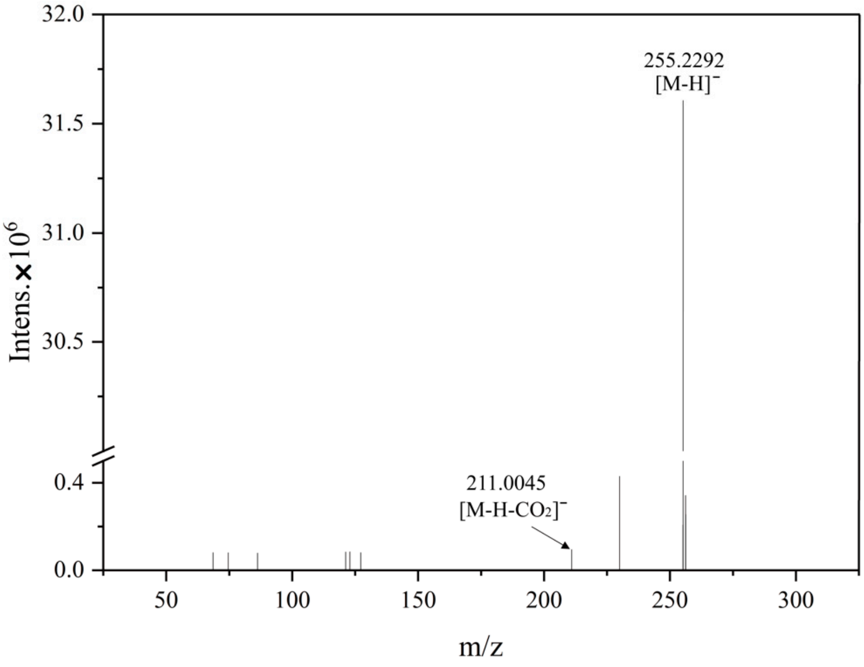

Figure S3. MS/MS spectrum of the OTA degradation product.

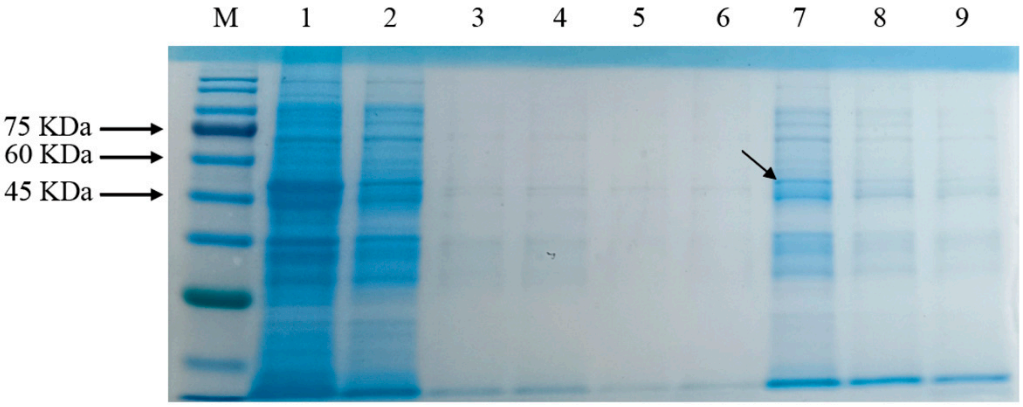

Figure S4. SDS-PAGE analysis of WGU28473.1. Lane M, molecular weight marker; Lane 1, cell lysate of *E. coli* BL21 (DE3)-pET-WGU28473.1 without IPTG; Lane 2, fiow through; Lane 3-5, wash 1-3; Lane 6-9, elution 1-4. Color arrows indicate the positions of protein bands corresponding to molecular weights of 75 kDa, 60 kDa, and 45 kDa.

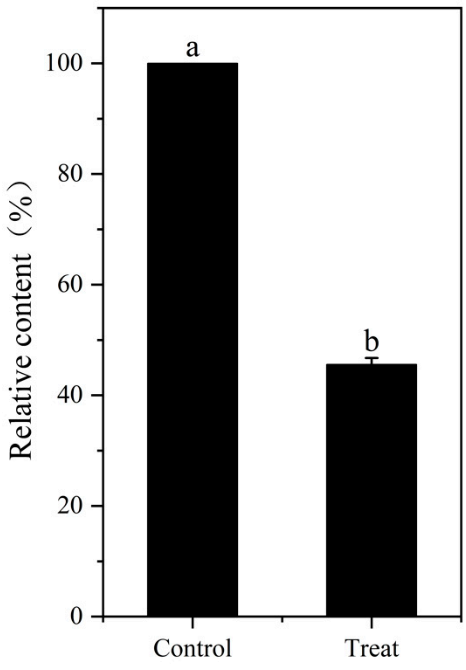

Figure S5. OTA degradation activity of purified WGU28473.1. The relative OTA content (%) is shown for the control (without protein) and the treatment with purified WGU28473.1. Data are presented as mean ± SD (n = 3). Different letters above the bars indicate significant differences ($p < 0.05$) between groups.
